# Microbiological evaluation of the steam sterilization of assembled laparoscopic instruments^1^

**DOI:** 10.1590/1518-8345.1431.2830

**Published:** 2016-11-21

**Authors:** Tamara Carolina de Camargo, Kazuko Uchikawa Graziano, Alda Graciele Claudio dos Santos Almeida, Karina Suzuki, Cely Barreto da Silva, Flávia Morais Gomes Pinto

**Affiliations:** 2 PhD, Assistant Professor, Faculdade de Ciências Médicas e da Saúde, Pontifícia Universidade Católica de São Paulo, Sorocaba, SP, Brazil.; 3 PhD, Full Professor, Escola de Enfermagem, Universidade de São Paulo, São Paulo, SP, Brazil.; 4 MSc, Doctoral student, Escola de Enfermagem, Universidade de São Paulo, São Paulo, SP, Brazil.; 5 PhD, Adjunct Professor, Faculdade de Enfermagem, Universidade Federal de Goiás, Goiânia, GO, Brazil.; 6 MSc, Pharmacy-Biochemistry, Santa Casa de Misericódia, São Paulo, SP, Brazil.; 7 PhD, Professor Instructor, Faculdade de Ciências Médicas, Santa Casa de Misericórdia, São Paulo, SP, Brazil.

**Keywords:** Sterilization, Laparoscopy, Surgical Instruments, Operating Room Nursing, Evidence-Based Nursing, Nursing

## Abstract

**Objective:**

assess the safety of steam sterilization of assembled laparoscopic instruments with challenge contamination.

**Method:**

a laboratory experimental study, using as test samples trocars and laparoscopic graspers. Geobacillus stearothermophillus ATCC-7953 was used, with a microbial population of 106UFC/Filter paper substrate, removed from the biological indicator. Three of them were introduced into each instrument at the time of assembly, and sterilized at pressurized saturated steam, 134oC for 5 minutes. After sterilization, the instrument was disassembled and each filter paper substrate was inoculated in soybean casein culture and incubated at 56oC for 21 days. In case of absence of growth, they were subjected to heat shock of 80oC, for 20 minutes and re-incubated for 72 hours. Sample size: 185 graspers and 185 trocars, with 95% power. We paired the experiments with comparative negative control groups (5 graspers and 5 trocars with challenge contamination, sterilized disassembled) and positive control (30 filter paper supports, unsterilized), subject to the same incubation procedures.

**Results:**

there was no microbial growth in experimental and negative control. The results of the positive control were satisfactory.

**Conclusion:**

this study provided strong scientific evidence to support the safety of steam sterilizing of the assembled laparoscopic instrument.

## Introduction

The videolaparoscopic surgery is a technological innovation that has emerged as an alternative to surgical, diagnostic and therapeutic procedures, which were usually performed through laparotomy. This technique has indisputable advantages for the patients, and new challenges for the nurses responsible for the Sterile Supply Center (SSC), including the establishment of guidelines for the safe processing of instruments and accessories with complex conformation, understood as those with less than 5 mm of lumen or blind-end, inaccessible internal spaces for direct friction, holes or valves^1^.

On the issue of sterilization, the pressurized saturated steam is the preferred method for heat resistant laparoscopic instrumental because it brings advantages such as low D^*^ value, high diffusivity and penetration of the sterilizing agent, speed, atoxicity and lower cost^2^. In this process pressurized saturated steam in contact with the cold surface of the material disposed within the autoclave, undergoes condensation, releasing the latent heat of vaporization watering and simultaneously heating the material. This heat causes thermal coagulation of proteins and death of microorganisms, i.e. pressurized saturated steam sterilization is based on heat exchange between the medium and the object to be sterilized^3^.

The classical recommendations state that the heat resistant surgical instruments are to be open, disassembled and with the surfaces free for steam sterilization^2^ ^,^ ^4^ ^-^ ^5^, including the laparoscopic ones. There are other guidelines that do not emphasize this kind of care^1^ ^,^ ^6^. There is no doubt that autoclaving of disassembled materials through thermal conduction provides the best condition.

Among health professionals, there is a deep-rooted concept that to achieve the success of the sterilization by the saturated pressure steam autoclave, direct contact of steam with all surfaces of materials is necessary, without considering the physical principles of latent heat. There is a need to question rooted concepts based on traditions, and strong scientific evidence should be sought in order to support decision-making in healthcare practice.

As laparoscopic accessories are complex instruments with several pieces of small size, if sterilized when completely dismantled, they can present problems for the surgical teams at the time of assembly in the operative field. It is noteworthy that some surgical scrub aides are unaware of the correct assembly, compromising their functionality, creating stress and disrupting the start of the surgical procedure.

The autoclaving of preassembled laparoscopic instruments is a reality identified by a survey, with a sample of 263 nursing professionals, in which 37% of respondents reported that in their institutions they sterilized the assembled laparoscopic instruments^7^. This practice aims at the optimization of time and security in the assembly process, but, on the other hand, there are surgical teams who question the SSC nursing team, asking if the sterilization through pressurized saturated steam of the assembled laparoscopic instruments is safe because it goes against the classical recommendations.

The scientific literature does not provide a conclusive answer about the safety of saturated steam pressure sterilization, of the assembled laparoscopic instrument ^8^ ^-^ ^10^, and it recommends conducting a new laboratory experimental test study^11^. To have an updated view we consulted the following portals and electronic databases on May 2016: PUBMED, BVS, EMBASE, SCOPUS e WEB OF SCIENCE, using the Boolean AND operator and controlled descriptors Medical Subject Headings (MeSH) steam, sterilization, laparoscopy e instruments. Three already known old articles were found^8^ ^-^ ^10^, published in the years 1991, 1995 and 2011, with no recent publications on the subject studied.

Given the abovementioned result, this research aimed to evaluate the safety of steam sterilization, of the assembled laparoscopic instrument with challenge infection, in order to bring to the table robust scientific evidence to support the decisions of the nurses that manage the SSC, focusing on the safety of the surgical patients.

## Method

This study is characterized as laboratory-based experimental. As test specimen we selected two types of reusable laparoscopic instruments that are more complex for reuse: Trocar with screw windowed valve, made of five parts, being one of them with a lumen of 5 mm diameter and 5 mm Dissection Forceps made of four parts with teethed end, lumen of 30 cm long and internal diameter of 3mm. The laparoscopic instruments used in the research were specific for this purpose and were not previously used in humans.

The selected challenge microorganism was the *Geobacillus stearothermophillus* ATCC-7953 in sporulated form, a biologic indicator available in the market to monitor steam pressurized sterilization cycles (Attest^TM(r)^ biologic indicator, reference 1.262, readings after 48 hs, steam). The self-contained biologic indicator is built in a paper substrate (2.5X0.5 cm) with a minimum of 100,000 calibrated dry spores of *Geobacillus stearothermophillus* ATCC-7953. We chose this microorganism because it is the standard for biologic monitoring of the efficacy control in autoclave cycles, due to its resistance to humid heat and low pathogenic conditions under normal conditions^12^.

Three groups were defined: an experiment group, a negative control and a positive control. In the experimental group we analyzed the results of microbial culture from 370 assembled laparoscopic instruments, coming from 185 graspers and 185 trocars, for a total of 1080 sampling units. This sampling size showed to have a 95% sample power, in which the chance of the assembled instrument of presenting viable spores after sterilization is at least 8%. As negative control group, 10 disassembled laparoscopic instruments were analyzed, composed of 5 graspers and 5 trocars, for a total of 30 sampling culture units. The positive control was a set of 30 non-sterilized paper filter substrate, inoculated seeding directly in TSB, 56^o^C for 48 hours. 

**Figure 1:**
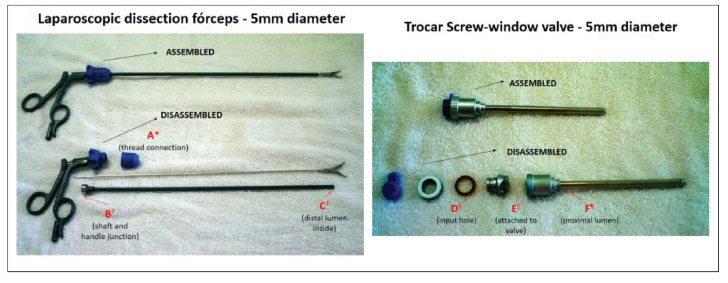
Placement of biologic indicators in the assemblage of laparoscopic instruments in positions A, B and C. Sao Paulo, SP, Brazil, 2014

The biologic indicator small tubes were disassembled using an aseptic technique, and the substrate papers with the *Geobacillus stearothermophillus* ATCC-7953 were separated. Three units of paper substrate were placed inside of each laparoscopic instrument in the process of assemblage (Figure 1). 

*Position A: thread connection of the laparoscopic dissection forceps. †Position B: Shaft and handle junction of the laparoscopic dissection forceps. ‡Position C: Distal lumen inside of the laparoscopic dissection forceps. §Position D: Trocar input hole with screw-window valve. ǁǁPosition E: Attached to trocar with screw-window valve. ¶Position F: proximal lumen of trocar with screw-window valve.

The instruments were packed individually in surgical grade paper and autoclaved in pressured saturated steam, with pre-vacuum autoclave Cisa^(r)^ model 6412HF, 558 liters, micro processed, thermal qualification for sterilization of surgical material at 134 C^o^ for 5 minutes.

After sterilization, the instruments were disassembled inside the biologically protected cabinet using aseptic techniques, and each biologic indicator paper substrates was seeded in *Tryptic Soy Broth* (TSB) culture medium, incubated at 56^o^ C for 21 days. If no microbiologic growth was observed after this time, tubes were exposed to a thermal shock during 20 minutes at 80^o^ C, re-incubating for 72 hs. at 56^o^C for a final reading^13^. This final process aimed to stimulate germination of spores that may have survived to autoclave.

The TSB culture medium that were used in the experiment were prepared form dehydrated media, as per manufacturer recommendation^14^. As and sterilization control of culture medium, 5% of the tubes were incubated at 36 ^o^ C during 7 days^15^. No microbiologic growth occurred in the samples.

## Results

The results of the experiments are presented in Table 1. Positive controls showed satisfactory growth confirming the challenge in the experiments, as well as the viability of the culture media and the adequacy of the incubation conditions for spore germination.

**Table 1:**
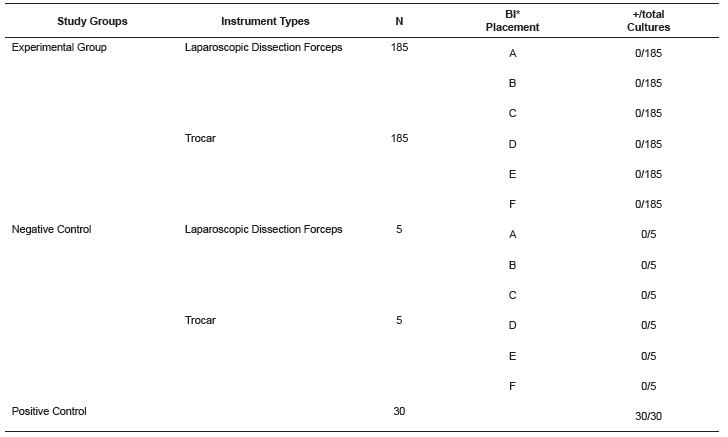
Results of the culture of paper substrate impregnated with spores coming form the Biologic Indicators (BI), inserted in the laparoscopic instruments assembled prior to sterilization (Experimental Group), of the Negative Control and of the Positive Control. Sao Paulo, SP, Brazil, 2013

* BI (Biologic Indicator).

## Discussion 

The present laboratory controlled research succeeded in sterilizing assembled laparoscopic instruments in pressurized saturated steam, thus proving the microbiologic safety of this procedure. An thermally qualified autoclave was used following the recommended parameters for pressurized saturated stem with pre-vacuum, at 134^o^C for 5 minutes^2^ ^,^ ^4^, associated to challenge contamination with spores *Geobacillus stearothermophillus* ATCC-7953 in three times 10^6^ UFC concentration, sterilization tests with direct inoculation method and a sample size that demonstrated robust results.

Autoclave sterilization of assembled laparoscopic instruments is a reality in Brazilian healthcare facilities^7^, against the classic recommendations that mandate the disassembling and opening of surgical instruments, exposing free surfaces to sterilization^2^ ^,^ ^4^ ^-^ ^5^. Results for the present research brings up strong scientific evidence of the safety of using pressurized saturated steam for sterilizing assembled laparoscopic instruments, supporting the regular practice in Brazilian institutions. The provision of pre-assembled laparoscopic instruments by the SSC's is an important facilitator and accelerator of the beginning of surgical procedures.

Sterilization of assembled laparoscopic instruments was studied previously^8^ ^-^ ^10^, concluding both positive and negative related to the practice of autoclaving assembled instruments, in spite of methodological issues arising form several of these papers.

The first research^8^ proposed the hypothesis that the assembled laparoscopic instrument would have the same sterilization safety compared to the disassemble instrument, using vegetative bacteria suspension (*Serratia marcescens*) and sporulated bacteria (*Bacillus subtilis e Bacillus stearothermophilus*) as challenge contamination of two laparoscopic graspers and two trocars (5mm and 10mm respectively). The inoculation technique and retrieval was done through swabs, retrieving the challenge microorganism both in assembled and disassembled laparoscopic sterilized instruments. In spite of the fact that the swab technique allows for a quantitative evaluation, it has limitations in standardizing the rolling resistance, the angle and the pressure degree during the procedure, it is not able to control reproducibility, and results have a large degree of variability^16^.

In this same research^8^ the authors question the fact of not having success in sterilization with disassembled instruments; something generally considered a best autoclaving practice. In the present research, we have succeeded in sterilization with pressurized saturated steam both of assembled and disassembled laparoscopic instruments. Worth of note is the methodological rigor, the 95 % sample power, the challenge contamination with spores of *Geobacillus stearothermophilus* in far higher concentration than the concentration found in worst case scenarios in clinical practice, associated with direct inoculation sterilization tests, ensuring full retrieval of viable microorganisms, respecting the incubation timing to allow the possible surviving spores may germinate after the thermal shock physical stimulus.

A different research^9^ used one of the parts of the laparoscopic instrument, a 12mm trocar with its lumen filled with organic material (hamburger meat) and microbial challenge contamination to assess the efficacy of sterilization using 132^o^ C in conventional and flash cycles with exposures of 10 and 3 minutes respectively. All vegetative microorganisms were eliminated with conventional and flash cycles of sterilization. Filling of the lumen with organic material showed resistance to direct steam contact, similar to the case when the laparoscopic instruments are sterilized while assembled.

In the same conditions, with organic material as lumen filling ^9^ researchers tested commercial biologic indicators *Geobacillus stearothermophilus* ATCC 7953 in the trocar lumen without hamburger meat and different time exposures, 3, 4,5 and 6 minutes. Only when time exposure was extended from 7 to 10 minutes the spores were fully destroyed. These results are in favor of the latent heat microbial destruction, in spite of the hard scenario of challenge contamination and massive organic material.

As the standard parameters for pressurized saturated steam with pre-vacuum autoclave are 134^o^ C in 4 minutes^2^ ^,^ ^4^ the researchers' need ^9^ of extending the sterilization time to succeed in fully eliminating the test microorganisms may be linked to the high concentration of organic material used in filling the trocar lumen, and not necessarily to the fact of the assembled trocars. The present research used the same microbiological challenge and succeeded in destroying the spores *Geobacillus stearothermophilus* ATCC 7953 using pressurized saturated steam with pre-vacuum sterilization cycle at 134 ^o^ C in 5 minutes.

Another research^10^ that assessed the efficacy of sterilization of single-use laparoscopic instruments, used as comparison group 50 reusable equivalent instruments that were autoclaved assembled. The challenge contamination was *Geobacillus stearothermophilus* ATCC 7953 with 10% of lamb blood. Instruments passed though automated cleaning in ultrasonic washer with intermittent flow and hand cleaning before assembling and sterilization under pressurized saturated steam with pre-vacuum at 134134 ^o^ C in 5 minutes. No microorganisms were retrieved in this group, reinforcing the chance of safety of sterilization under pressurized saturated steam of assembled instruments.

Researchers^10^ through instrument cleaning had certainly reduced contamination, thus being impossible to quantify the real challenge imposed in the experiment to asses the assembled instrument sterilization. In the case of our research, three units of substrate paper impregnated with *^_Geobacillus stearothermophilus 106 UFC_^* were placed inside each laparoscopic instrument before sterilization, thus creating a three fold 10^6^ UFC challenge of the test microorganism in each sample unit.

## Conclusion

Sterilization under pressurized saturated steam of assembled laparoscopic instruments is microbiologically safe, breaking with the paradigm of classic recommendations of autoclaving only disassembled material. Results of this research, under the experiment conditions, are strong scientific evidence that supports a systematic review of this topic and gives inputs to the decision-making process related to the microbiological safety of pressurized saturated steam sterilization of assembled laparoscopic instruments. Additionally, it is desirable that it may give inputs to lawmakers to formalize the possibility of autoclaving pre-assembled laparoscopic instruments.

## Footnotes

* D (decimal reduction time): is a time interval at constant temperature needed to reduce in 90% the starting microbial population(2).

## References

[1] Ministério da Saúde (BR). Agência Nacional de Vigilância
Sanitária (2012). Resolução da Diretoria Colegiada n. 15, de 15 de março de 2012. Dispõe sobre
requisitos de boas práticas para o processamento de produtos para saúde e dá
outras providências.

[2] Rutala WA, Weber JD (2008). Healthcare Infection Control Practices Advisory Committee (HICPAC). Guideline
for disinfection and sterilization in healthcare facilities.

[3] Francis MJ, Pashley RM (2009). Application of a bubble column for evaporative cooling and a simple
procedure for determining the latent heat of vaporization of aqueous salt
solutions. J Phys Chem B.

[4] American National Standard (2006). Association for the Advancement of Medical Instrumentation. Comprehensive
Guide to Steam Sterilization and Sterility Assurance in Health Care
Facilities.

[5] Committee on Infection Control in the Handling of Endoscopic
Equipment (1980). Guidelines for preparation of laparoscopic
instrumentation. AORN J.

[6] Association of Perioperative Registred Nurse (2016). Guidelene for Sterilization. Guideline for perioperative practice.

[7] Camargo TC, Feitosa AS, Graziano UK (2014). Identificação e análise da prática de esterilização do instrumental
laparoscópico montado. Rev SOBECC.

[8] Marshburn PB, Rutala WA, Wannamaker NS, Hulka JF (1991). Gas and steam sterilization of assembled versus disassembled
laparoscopic equipment. Microbiologic studies. J Reprod Med.

[9] Voyles CR, Sanders DL, Simons JE, McVey EA, Wilson WB (1995). Steam sterilization of laparoscopic instruments. Surg Laparosc Endosc.

[10] Lopes CLBC, Graziano KU, Pinto TJA (2011). Evaluation of Single-use Reprocessed Laparoscopic Instrument
Sterilization. Rev. Latino-Am. Enfermagem.

[11] Camargo TC, Rocha CDA, Graziano KU (2008). Steam sterilization of previously-assembled laparoscopic
instruments. Acta Paul Enferm.

[12] Albert H, Davies DJG, Woodson LP, Soper CJ (1998). Biological indicators for steam sterilization: characterization of a
rapid biological indicator utilizing Bacillus stearothermophilus spore-associated
alpha-glucosidase enzyme. J Appl Microbiol.

[13] Sterility test (2008). The United States Pharmacopeia.

[14] Zimbro MJ, Power DA, Miller SM, Wilson GE, Johnson JA (2009). Manual of Microbiological Culture Media.

[15] Arghyros M, Douglass G, Löcher M, Mugg P, Myatt DC, Olma T, Scholtes A, Wilkinson I (2012). Guidelines for assuring quality of medical microbiological culture
media.

[16] Moore G Griffith (2007). Problems associated with traditional hygiene swabbing: the need for
in-house standardization. J Applied Microbiol.

